# Differential gene expression in peripheral vascular smooth muscle cells of patients with peripheral artery disease

**DOI:** 10.1007/s10157-025-02774-w

**Published:** 2025-10-22

**Authors:** Dongoh Lee, Ji Hye Kim, Dong Yeon Lee

**Affiliations:** 1 SNU Seoul Hospital, Seoul, Korea; 2 Department of Orthopedic Surgery, Seoul National University Hospital, College of Medicine, Seoul National University, 101 Daehak-No, Jongno-Gu, Seoul, 110-744 South Korea

**Keywords:** Vascular smooth muscle, Peripheral artery, Transcriptome, Vascular calcification, Chronic kidney disease

## Abstract

**Background:**

The role of peripheral vascular smooth muscle cells (VSMCs) in vascular calcification has been overlooked compared with that of the major VSMCs. This study aimed to investigate the differentially expressed genes (DEGs) of peripheral VSMCs in patients with critical limb ischemia (CLI) results from peripheral arterial disease and Chronic Kidney disease (CKD).

**Methods:**

We isolated peripheral VSMCs from the posterior tibial artery of 6 patients with CKD who underwent below-knee amputation for CLI. Using normal human aortic VSMCs as a control, we cultured all samples in normal and high phosphate media for 10 days. Total RNA was extracted and analyzed using mRNA sequencing. Expression levels of genes related to contractile and synthetic phenotypes were examined. Bioinformatics analysis of the DEGs was performed.

**Results:**

All four genes (*ACTA2, CALD1, CNN1,* and *TAGLN*) related to the contractility phenotype increased only in the control group. The expression of all four genes (*ICAM1, SPP1, MMP3,* and *TIMP1*) related to the synthetic phenotype showed no significant changes or decreases in all samples. Several genes (*SERTAD4, ITGA11, SPRN, IGFBP6, BCL2A1, APOE, TRABD2A,* and *FAM13B*) showed significant changes under calcifying conditions. Only *UNC5B* expression showed an opposite pattern between normal human aortic VSMC and pathological peripheral VSMCs.

**Conclusions:**

*UNC5B* was overexpressed only in pathologic peripheral VSMCs under calcifying conditions, whereas downregulated in normal aortic VSMCs. Further research on the effect of *UNC5B* on peripheral VSMC is warranted. (IRB number: H-1711-022-897).

## Introduction

Vascular smooth muscle cells (VSMCs) play active roles in vascular calcification [1, 2]. Vascular calcification does not occur singly; it is a complex pathological process by VSMCs, which may differ according to its histological location in the blood vessel and anatomical location in the arterial tree [2]. A recent study demonstrated that the biology of arterial beds, and thus the involvement of VSMCs in vascular calcification, differs between anatomical sites such as the carotid and coronary arteries [3].

Peripheral artery disease (PAD) is caused by insufficient blood flow to the limbs due to occlusion or narrowing of peripheral arteries. Chronic kidney disease (CKD) is a significant risk factor for PAD which can lead to debilitating conditions such as critical limb ischemia (CLI), in which patients may require amputation [4]. The limited options for the treatment of PAD are mechanical modalities, such as endovascular stent therapy. Vascular calcification acts as a potential driver of PAD; it is a risk factor for restenosis in endovascular stent therapy and increases the risk of limb amputation [5].

However, the literature on the role of VSMCs has mainly been based on major arteries rather than peripheral arteries. Notably, PAD is not less clinically significant than diseases of major vessels such as coronary artery disease [3, 6]. Surprisingly, studies regarding the calcifying characteristics of peripheral VSMCs in CKD with CLI have been limited in both the number and scope [7].

Understanding the molecular and cellular processes that accelerate vascular calcification may facilitate the development of preventive pharmacotherapy and thus have a tremendous impact on cardiovascular disease and PAD [8]. We posited that an exploration of the genes that may aggravate or prevent peripheral vascular calcification in pathologic states could be the key to advancing the prevention of peripheral vascular calcification by using medical modalities such as gene therapy.

Thus, the purpose of this study was to compare differential gene expression in peripheral VSMCs cultured in a calcifying medium and a general medium using RNA sequencing analysis. We hypothesized that some genes or gene sets possibly participate in the pathological process of peripheral vascular calcification in patients with CKD.

## Materials and methods

This study was approved by the institutional review boards of Seoul National University Hospital and Myongji Hospital, Hanyang University College of Medicine. Informed consent was obtained from all the participants in this study, which was performed in accordance with the principles outlined in the Declaration of Helsinki.

Patients who fulfilled the following criteria were included in the study: (1) patients with CLI who showed necrosis of the foot and required knee amputations for severe PAD; (2) patients with CKD who had an estimated glomerular filtration rate < 60 ml/min/1.73 m^2^; and (3) patients with vascular calcification in the lower leg as diagnosed by simple radiographs. We excluded patients who (1) had a history of a medial ankle injury from which we would harvest or (2) had other diseases, such as vasculitis, which could affect the outcome of vascular biopsy.

The 10 samples were obtained from 10 patients (*n* = 10) who underwent below-knee amputations at either Seoul National University Hospital (*n* = 8) or Myongji Hospital, Hanyang University College of Medicine (*n* = 2).

### Isolation and culture of human VSMCs

Vascular biopsies showing pathologies in the amputated legs of the patients were obtained at the time of surgery. For the isolation of pathological peripheral VSMCs, we excised approximately 5 cm of the posterior tibial artery from the amputated ankle. Isolation and culture of VSMCs were performed using the enzymatic tissue digestion method [3].

Human vascular aortic smooth muscle cells (catalog no. C-12533) were purchased from PromoCell (Heidelberg, Germany) or isolated from the human aorta using an in-house protocol. In brief, arterial tissues were removed from the adventitia, and the endothelium was scraped. Arterial strips were washed thrice with 1 × phosphate-buffered saline to remove the blood and then were digested using 2 mg/mL collagenase type II enzyme (Sigma-Aldrich) for 1 h at 37 °C in Dulbecco’s modified Eagle medium (Gibco, NY, USA). Next, the obtained cell suspension was centrifuged, and the pelleted VSMCs were collected and seeded in 6-well plates containing smooth muscle cell growth medium (SMC-GM2; PromoCell, Heidelberg, Germany), to which 1% antibiotic–antimycotic (Gibco) was added.

Seeded cells were incubated at 37 °C in a humidified atmosphere containing 5% CO_2_. VSMCs were propagated during subsequent passages in SMC-GM2 with an average incubation time of 7 d. Cell viability was assessed using trypan blue staining after each passage. All assays were conducted with subconfluent cells from passages 1–3 [9]. For the calcification experiments, VSMCs from both groups were grown using different media depending on the experiment; for the control group, smooth muscle cell growth medium was used, and inorganic phosphate (Pi) concentrations were adjusted using stocks of 3 mM Na_2_HPO_4_/NaH_2_PO_4_ (pH 7.4) [9, 10]. We changed the media every 2 d in all cultures.

### Next-generation sequencing

The total RNA was extracted using an RNeasy Mini Kit (Qiagen, Valencia, CA, USA) according to the manufacturer’s instructions. RNA sequencing was performed by EBIOGEN Inc. (Seoul, South Korea). In brief, RNA quality control was measured using an Agilent 2100 bioanalyzer with an Agilent RNA 6000 Nano kit (Agilent Technologies, Amstelveen, Netherlands). RNA was quantified using an ND-2000 spectrophotometer (Thermo Fisher Scientific, Wilmington, DE, USA). Libraries were constructed using the QuantSeq 3′ mRNA-Seq Library Prep Kit (Lexogen, Vienna, Austria). High-throughput sequencing was performed as single-end 75 sequencing using a NextSeq 500 (Illumina, Inc., San Diego, CA, USA).

### Identification of differentially expressed genes (DEGs)

Analysis of DEGs and gene ontology was performed using the Excel-based Differentially Expressed Gene Analysis (ExDEGA) software program (EBIOGEN, Seoul, Korea) [11].

Before screening candidate genes that may be involved in the pathologic process of vascular calcification, we identified expression levels of known genes related to contractile and synthetic phenotype from all samples in patients with CLI [3]. Primers for genes related to contractile phenotype were α-smooth muscle actin (ACTA2), h-caldesmon (CALD1), calponin 1 (CNN1), and transgelin (TAGLN); for synthetic phenotype, they were myosin, intercellular adhesion molecule 1 (ICAM1), secreted phosphoprotein 1 (SPP1, also osteopontin), matrix metallopeptidase (MMP) 3, and tissue inhibitor of metallopeptidase 1 (TIMP1).

Candidate genes were then explored from the samples using the aforementioned tools. Genes expressed with a greater than twofold change cultured in high phosphate media compared with cells cultured in general media and with a *p* value < 0.05 were considered DEGs, as previously described [11]. We also explored genes that were significantly increased or decreased by more than 1.5-fold in their expression [12].

### Bioinformatics analysis

Scatter plot analysis, heatmap generation, clustering analysis, and gene plot analysis for the selected genes were performed using ExDEGA. Ontology sets (c5.all.v7.4. symbols.gmt) were generated using the Gene Set Enrichment Analysis (GSEA) 4.1.0 program [13]. The false discovery rate was set as *q* < 0.05 [14].

### Quantitative real-time PCR

RNA was isolated from cells using Qiagen RNA extraction kit. (Qiagen, Hilden, Germany), and cDNA synthesis was performed using the SuperScriptTM III First-Strand Synthesis System (Invitrogen, Waltham, MA, USA) according to the manufacturer’s instructions. Quantitative RT-PCR reactions were conducted in a LightCycler^®^ 480 Instrument II (Roche, Rotkreuz, Switzerland) in 20 µL reaction mixtures with SYBR Green PCR Master Mix kit (Thermo Scientific, USA). PCR were performed using specific primers for UNC5B, ACTA2, ICAM1 and GAPDH as control. Experiments were performed in triplicate.

### Statistical analysis

Quantified data are presented as the mean ± standard deviation. Each experiment was performed twice. Statistical significance was assumed at a 95% confidence level (*p* < 0.05). All statistical analyses were performed using SPSS version 19.0 (IBM Corp., Armonk, NY, USA).

## Results

The mean age of the patients was 71.4 years old (range, 56–86 years), and all were men. Six patients had diabetes, and four had cardiac vessel disease. One patient had chronic liver disease, and one patient had a history of cerebral infarction.

### DEG identification

Six pathologic samples were included in the analysis because the other four samples showed inconsistent outcomes in two repetitive sessions of RNA sequencing. Notably, the expression of all four genes (*ACTA2, CALD1, CNN1,* and *TAGLN*) related to the contractility of VSMC was significantly increased by more than 1.5-fold only in normal human aortic VSMC in calcifying media, and the expression of these genes showed no significant change or significantly decreased in the other pathologic peripheral VSMCs. The expression of all four genes (*ICAM1, SPP1, MMP3,* and *TIMP1*) related to the synthetic phenotype showed no significant change or was significantly decreased in all samples, including normal human aortic VSMC.

**Fig. 1:**
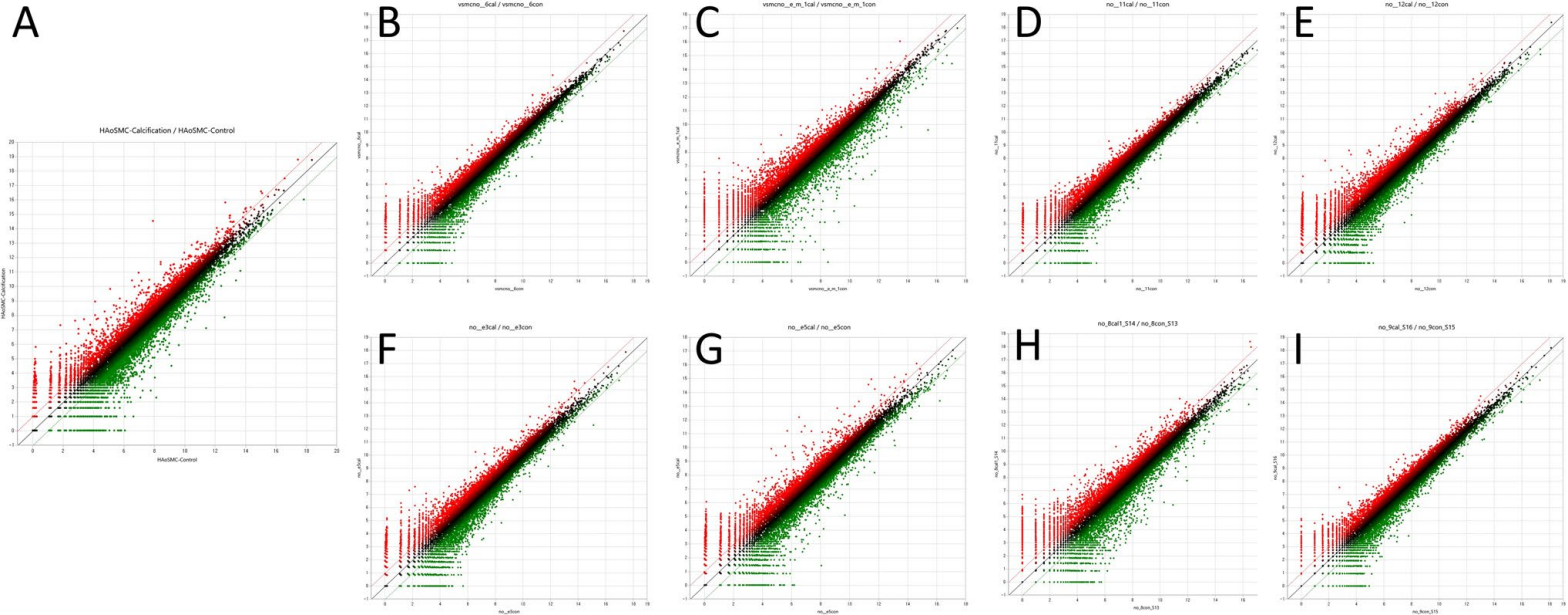
Scatter plots of gene expression. **a** Expression in human aortic VSMC as a control group. **b**-**i** Expression in each pathologic peripheral VSMCs. In each plot, the central line passing through the origin indicates no difference in expression between cells stored for different time periods. Red plots denote upregulated genes; green plots denote downregulated genes. Plots outside the dotted line (red or green) indicate a more than twofold difference

Next, we performed whole transcriptome RNA sequencing to identify the differences in gene expression profiles between groups cultured in calcifying media and those cultured in normal media. The scatter plot analysis showed that the DEG profiles of the samples overlapped considerably (Fig. 1). The DEG profiles of human aortic VSMCs responding to calcifying media compared with normal media were somewhat different from those of pathological peripheral VSMCs responding to calcifying media compared with normal media, although the difference was not statistically significant.

We detected only 2 genes, *SERTAD4* and *ITGA11*, which were expressed significantly more than twofold in cells cultured in calcifying media compared with cells cultured in general media. These two genes were overexpressed in all samples, with normal human aortic VSMC used as the control group.

In searching for genes expressed beyond a 1.5-fold change, we detected 17 genes. Of these, we excluded 10 genes because they showed conflicting expression patterns; these were overexpressed in some samples and decreased in others. We found seven genes (*UNC5B, SPRN, IGFBP6, BCL2A1, APOE, TRABD2A,* and *FAM13B*) that showed consistent patterns in all pathological peripheral VSMC samples. Among them, *UNC5B* genes were overexpressed in all pathologic peripheral VSMC samples, whereas they were suppressed in human aortic VSMC as a control group. The expression of five genes (*SPRN, IGFBP6, BCL2A1, APOE,* and *TRABD2A*) was decreased in all samples, including the control group, and *FAM13B* was overexpressed in all samples.

### Heatmap generation and clustering analysis

**Fig. 2:**
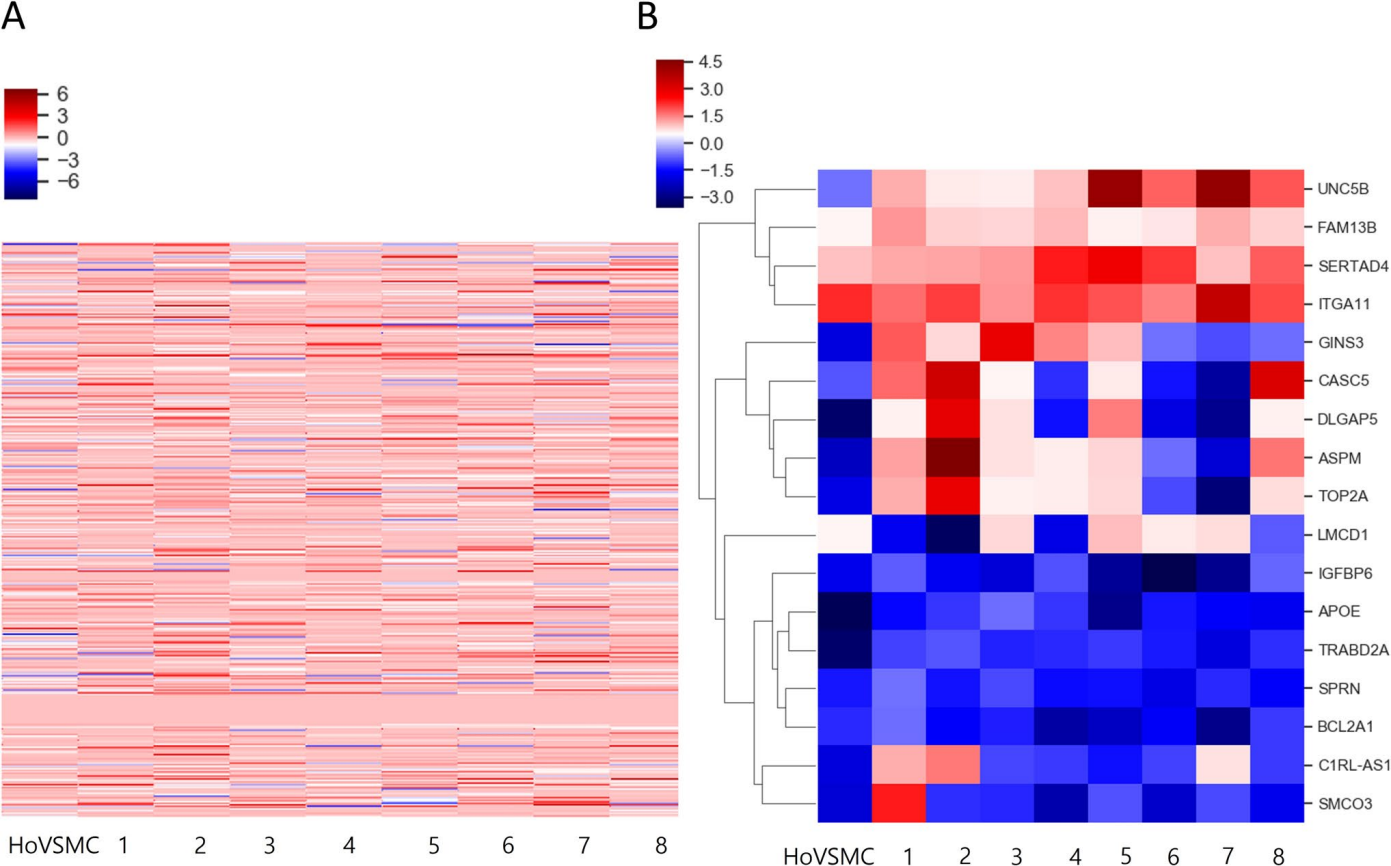
**a** Heat map and hierarchical clustering of the RNA sequencing data from all samples. **b** Clustered heatmap of significant transcriptional changes expressed beyond 1.5-fold change from samples; pathologic peripheral VSMCs and human aortic VSMC as a control group. This heatmap shows a clear specific expression profile comparing the calcifying condition and general condition

We found that the gene expression of normal human aortic VSMCs in calcifying media compared with that cultured in normal media showed global changes when compared with the gene expression of pathologic peripheral VSMCs in calcifying media compared with that cultured in normal media (Fig. 2).

### Gene plot analysis

**Fig. 3:**
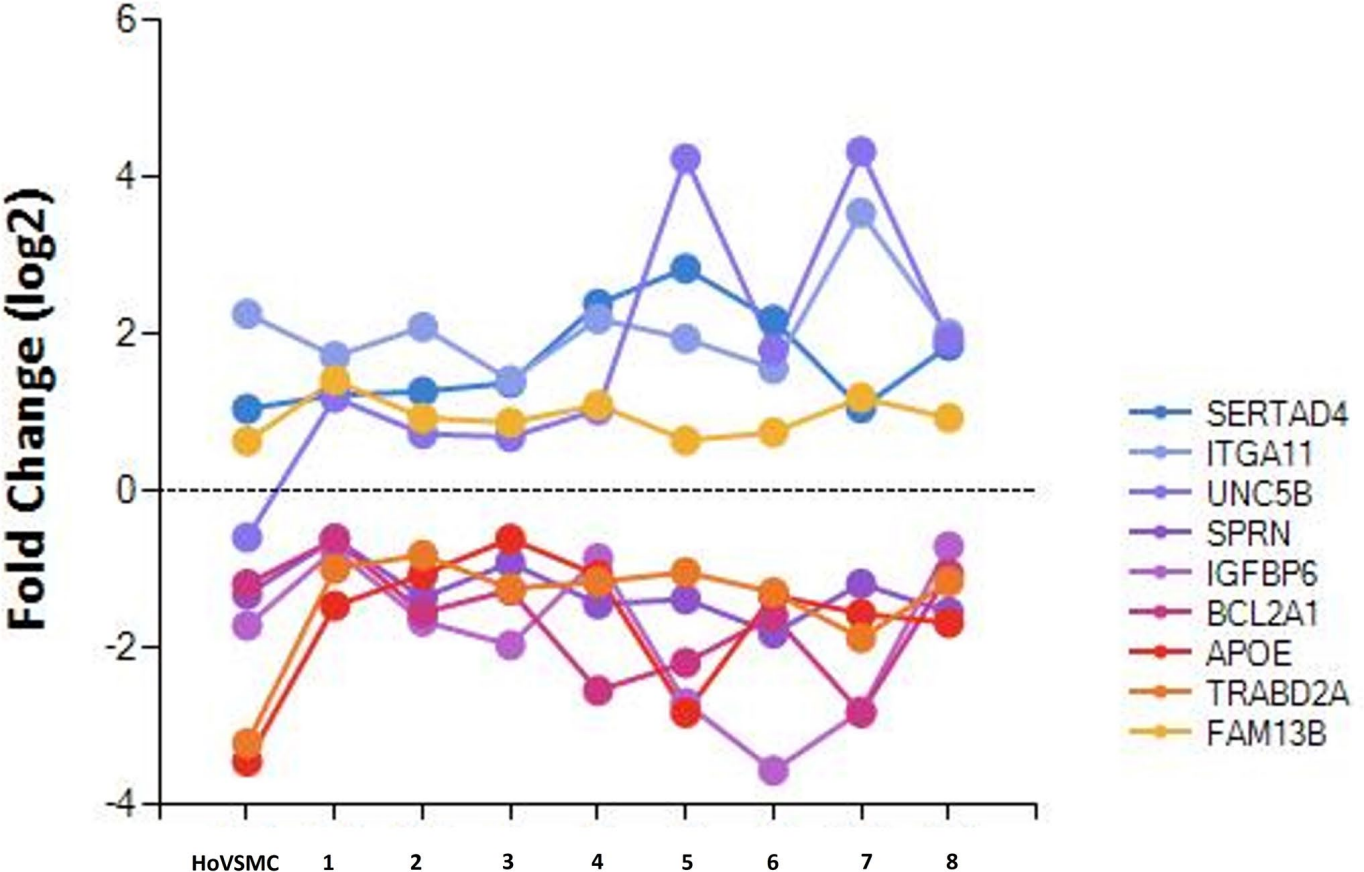
Gene plot analysis. Expression of selected 9 genes in gene screening was presented in all samples, including normal human aortic VSMCs as a control group. HoVSMC: normal human aortic VSMC

Several candidate genes with greater than twofold and 1.5-fold changes expressed in calcifying media compared with cells cultured in general media and with a *p* value < 0.05 were shown in the gene plot analysis (Fig. 3).

### Gene set enrichment analysis (GSEA)

**Fig. 4:**
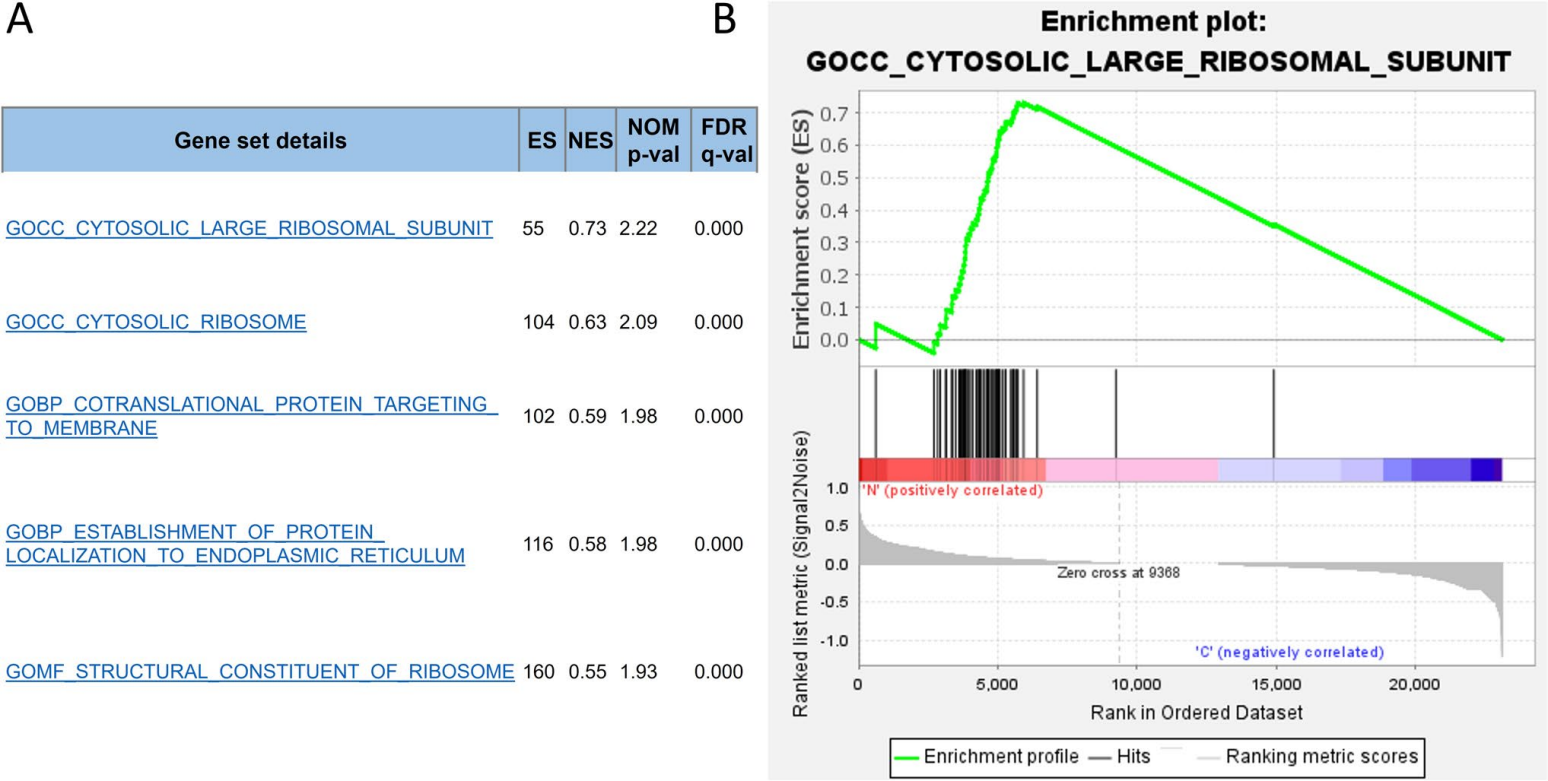
Gene set enrichment analysis. **a** Only gene sets with *q* value < 0.05 were included, all of which were from samples cultured in general media. No significant gene sets from samples cultured in calcifying media were screened in the c5 gene ontology set analysis. **b** One of 5 gene sets enrichment plots with significant *q* value from samples cultured in general media

Five significantly enriched gene sets from samples cultured in general media as a control group were screened: gene ontology of cytosolic large ribosomal subunit, cytosolic ribosome, contranslational protein targeting to membrane, protein localization to the endoplasmic reticulum, and structural constituent of ribosome (Fig. 4). No gene sets were enriched in samples cultured in calcifying media.

**Fig. 5:**
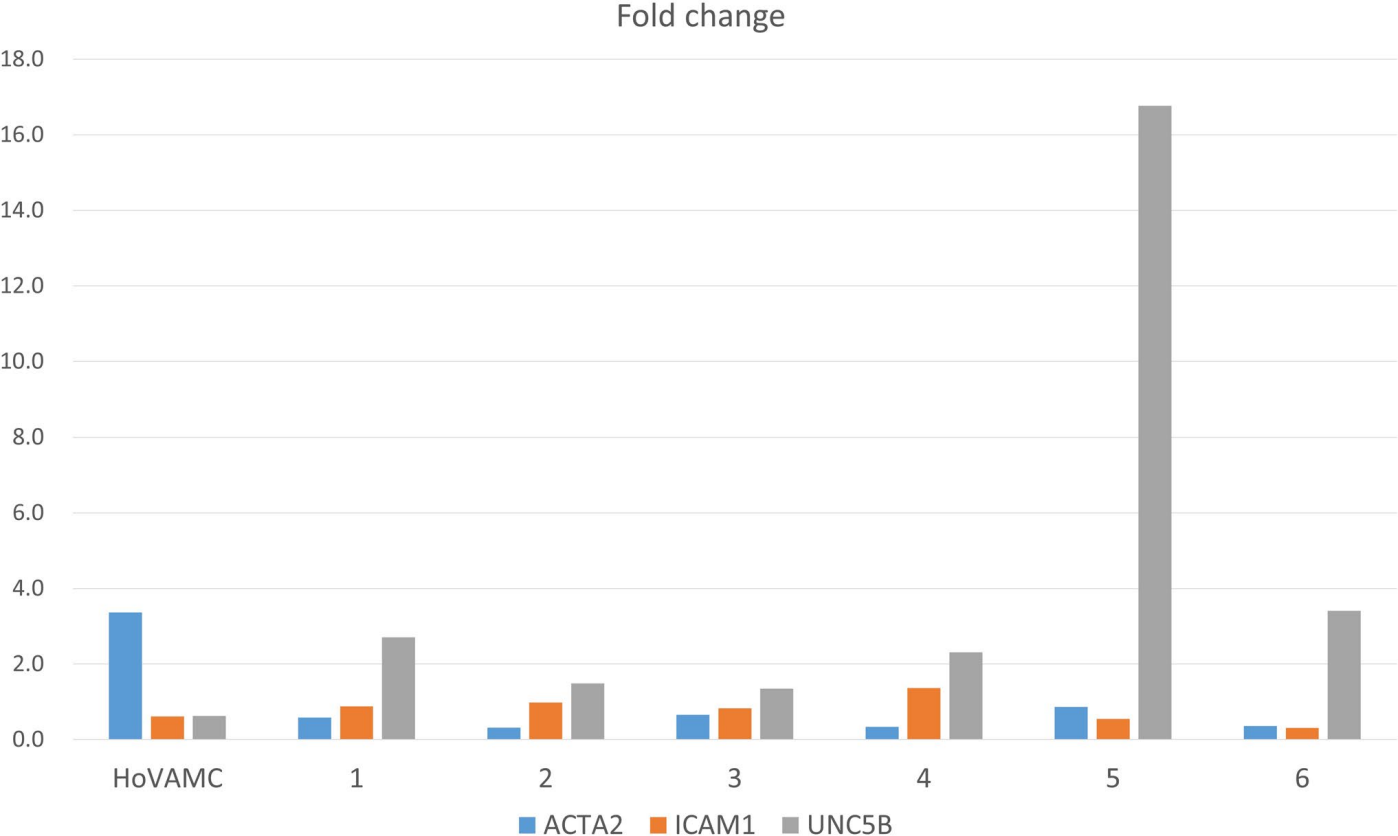
Expression of 3 genes (ATCTA2, ICAM1, UNC5B) in control cell and six samples using real-time PCR

We performed real-time PCR to validate the expression of UNC5B and other genes potentially related to vascular calcification (Fig. 5).

## Discussion

**Fig. 6:**
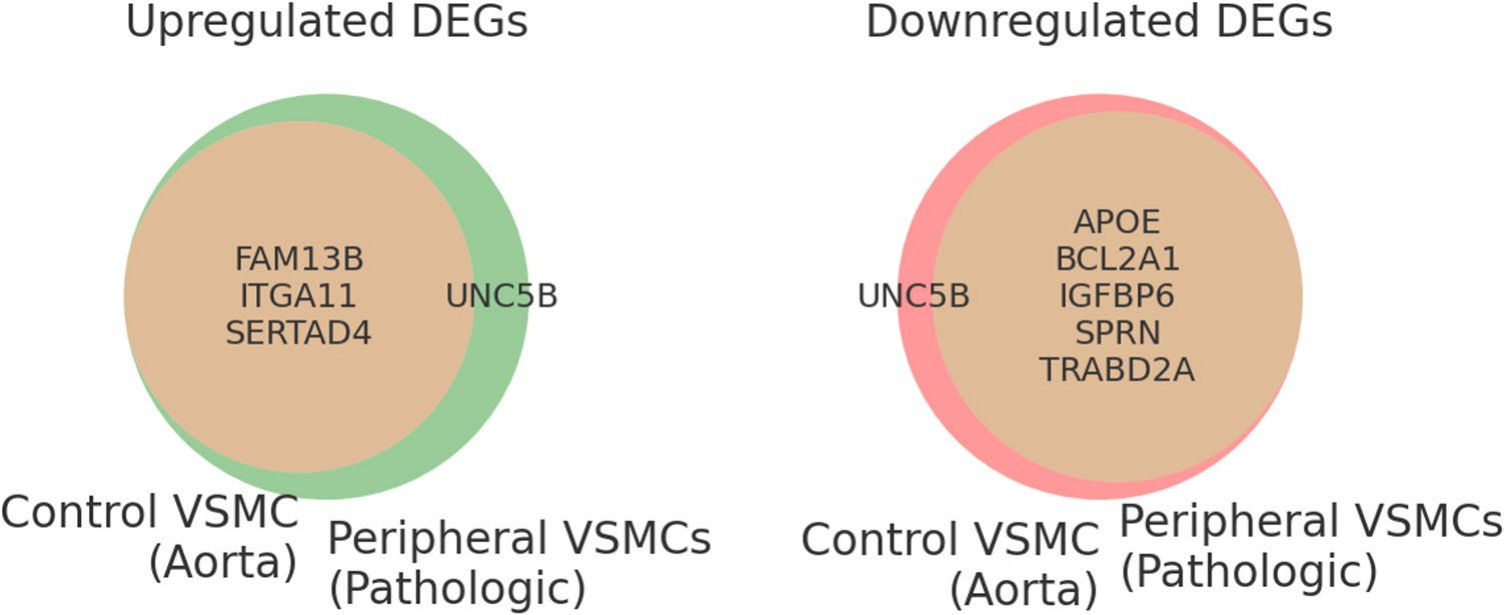
Venn diagrams to classify the differentially expressed genes

In this study, we have revealed that the pro-calcifying characteristics of VSMCs in patients with PAD were similar to those of in vitro studies reporting VSMCs in major vessels [4]. However, the degree of calcification in pathologic peripheral VSMCs was much greater than that in normal aortic VSMCs. We suggest nine candidate genes that may be related to peripheral vascular calcification in patients with CKD and CLI, using RNA sequencing and bioinformatics. The expression of two genes, *SERTAD4* and *ITGA11*, was significantly increased under calcifying conditions, regardless of normal major or pathologic peripheral VSMCs. *UNC5B* was exclusively overexpressed in pathological peripheral VSMCs. The expression of five genes (*SPRN, IGFBP6, BCL2A1, APOE,* and *TRABD2A*) was decreased in all samples, and *FAM13B* was overexpressed in all samples (Fig. 6).

Elevation of serum phosphate salt level is known to be a risk factor for vascular calcification in patients with CKD [15]. Calcification of VSMCs would be triggered by high phosphate treatment, the mechanism of which remains unclear [15]. High phosphate-inducing methods for the calcification of VSMC have been widely employed in studies regarding vascular calcification [16]. We also applied this method to analyze DEGs from VSMCs receiving calcifying stimuli.

VSMCs under high phosphate conditions dedifferentiate into a synthetic phenotype and facilitate vascular calcification. Many studies have explored various pathways involving this mechanism [17]. This study compared the behavior of VSMCs in general media and calcifying media in two types of cells (pathologic peripheral VSMC and normal major VSMC). We previously found that cells cultured from diseased peripheral VSMC had much more calcification than cells cultured from normal major VSMC, although the two groups had common features of VSMCs [18].

Before this study, we expected that the expression of genes related to contractility would be decreased and that genes related to the synthetic phenotype would be overexpressed under calcifying conditions [3, 19]. However, our results differed from our expectations. Human aortic VSMC, as a control sample, showed increased expression of genes related to the contractility phenotype, although this was not statistically significant. In addition, the expression of genes related to the synthetic phenotype of VSMC decreased or showed no significant change under calcifying conditions. There may be some type of defensive mechanism to calcify stimuli in normal aortic VSMC, which would be defective in pathological peripheral VSMCs.

The functions of specific genes detected in this study have not been fully elucidated in the literature. Proteins containing the SERTAD4 domain have been linked to cell cycle progression and chromatin remodeling [20]. *SERTAD4* is known to be expressed in many types of tissues. The degree of its expression was noticeable in the smooth muscles of the heart, smooth muscle, and skeletal muscle. One study suggested that this gene was one of the 1431 upregulated genes in the inhibition of atherosclerosis development; however, the authors could not determine its function in detail [21].

*ITGA11*, Integrin alpha 11, is mainly expressed in female genital organs and smooth muscle. Bansal et al. reported that *ITGA11* was co-localized with α-smooth muscle actin-positive myofibroblasts and was correlatively induced with increasing fibrogenesis in human fibrotic organs [22]. *ITGA11* knockdown dramatically altered the myofibroblast phenotype, including the impaired contractility of collagen I matrices in their study [22]. Our findings support those of their study, highlighting the significance of the ITGA11 receptor as a promising therapeutic target in organ fibrosis.

The expression of *UNC5B* showed an opposite pattern between normal human aortic VSMC and pathologic peripheral VSMCs in the analysis: decreased in human aortic VSMC and increased in pathologic peripheral VSMCs under calcifying conditions. One study suggested that the netrin-1 receptor, UNC5B, plays critical roles in cell survival and kidney injury [23]. The other studies suggested that UNC5B is related to angiogenesis in diabetic kidney disease [24].

Netrin-1 is widely expressed in the cells of the vascular endothelium, liver, and colon and is highly present in the kidneys [24]. Netrin-1 is involved in the control of angiogenesis; however, there are controversies over the role of this protein, and whether it exerts a pro or an anti-angiogenic effect remains unclear. UNC5B is the only netrin-1 receptor primarily expressed in the vascular system [25]. We suspect that UNC5B in pathologic peripheral VSMC, which was overexpressed in calcifying media, is related to PAD.

This study had several limitations. First, we could not compare cells from the same origin, which might have confounded our results. VSMCs from major vessels, such as the aorta of the same patient from whom we harvested peripheral arteries, could have enabled a more valuable analysis, although we could not obtain such samples because of ethical arguments. Another limitation of our study is the absence of a direct comparison between normal aortic VSMCs and pathological peripheral VSMCs under normal media conditions, which may have provided further insight into their baseline transcriptional differences. Second, the sample size in this study was relatively small. Therefore, further research with a higher number of samples than we used, including pathological and normal VSMCs, are required. Third, we did not provide the baseline characteristics of the patients in detail because we did not expect their characteristics could affect our results. Actually, all of patients had chronic renal failure, who underwent below-knee amputation. Hence, the overall characteristics may be expected to be similar between them.

## Conclusion

We demonstrated considerable changes in the transcriptional profile of pathological peripheral and normal aortic VSMC under calcifying conditions. Moreover, *UNC5B* was overexpressed only in pathologic peripheral VSMC under calcifying conditions, whereas it was downregulated in normal aortic VSMC.
